# Photoneutrons and Gamma Capture Dose Rates at the Maze Entrance of Varian TrueBeam and Elekta Versa HD Medical Linear Accelerators

**DOI:** 10.3390/toxics11010078

**Published:** 2023-01-14

**Authors:** Ibrahim I. Suliman, Ghada A. Khouqeer, Fareed H. Mayhoub

**Affiliations:** 1 Department of Physics, College of Science, Imam Mohammad Ibn Saud Islamic University (IMSIU), Riyadh 11642, Saudi Arabia; 2 Biomedical Physics Department, King Faisal Specialist Hospital & Research Center, Riyadh 11564, Saudi Arabia

**Keywords:** radiotherapy, X-rays, neutron contaminations, radiation dosimetry, radiation measurements

## Abstract

Herein, we evaluated the neutron and gamma capture dose equivalent rates at the maze entrance of Varian TrueBeam and Elekta Versa HD™ medical linear accelerators (linacs) using experimental measurements as well as empirical calculations. Dose rates were measured using calibrated neutron and gamma area survey meters placed side-by-side at the measurement point of interest. Measurements were performed at a source-to-detector distance of 100 cm, with a 10 × 10 cm^2^ field size therapeutic X-ray beam, and a 30 × 30 × 15 cm^3^ solid water patient equivalent phantom, with a linac operating at 15, 10 MV, and 10 MV flattened filter-free (FFF). Dose rates were also measured at different points at the centerline along the maze towards the maze entrance. The measured dose equivalent rates at the maze entrance were comparable to those reported in the literature. The dose rates along the maze decreased exponentially towards the maze entrance and were significant for short maze lengths. The evaluated empirical methods for estimating neutron dose rates at the maze entrance of a linac proposed by Kersey, the modified Kersey method and Falcão method, agree by a factor of two from the experimental measurements. The results revealed vital radiation protection considerations owing to neutron contamination in external beam therapy.

## 1. Introduction

Cancer growth occurs when malignant cells proliferate abnormally throughout the body, becoming one of the common causes of death worldwide [1,2]. Radiation therapy (RT) is an available cancer treatment. Other treatment options include surgery and chemotherapy. Radiotherapy using medical linear accelerators (linacs) is the most important cancer treatment modality in modern medicine [3,4]. In comparison to other treatment modalities, linacs reduce scattered radiation, otherwise damaging healthy tissues by having a low skin and high dose depth [5,6]. Hence, most of the radiotherapy treatment is performed using electrons and megavoltage X-rays from linacs [7].

High-energy electrons and photons create a variety of secondary particles after interacting with materials in the accelerator head, including targets, flattening filters, collimation systems, and other structural components [8]. Neutron production is important for radiation protection.

According to Mobley and Laubenste Ref. [9], in hydrogenous materials, the threshold for photoneutron production is 2.2 MeV for deuterium atoms. In radiotherapy using high-energy X-rays, significant neutron dose and presence were shown at 6 MV linac using the novel tensioned metastable fluid detector (TMFD) sensor technology [10]. In general, photoneutron intensity in photonuclear reaction (γ, n) increases with photon energy up to 25 MV [11].

Secondary neutrons in radiotherapy are formed through a photonuclear reaction (γ, n). Neutrons have a higher linear energy transfer (LET) than photons and thus have a high radiological risk [12,13] when the incident high energy X-ray photons interacts with the accelerator head materials Consequently, radiotherapy treatment rooms contaminated with photoneutrons can cause patients to receive excessive radiation and increase the risk of secondary cancer.

Radiation protection aims to protect humans and the environment from the harmful effects of ionizing radiation. Linear accelerators are potential sources of radiation hazards; which is why they are operated in shielded rooms. In general, shielding calculations for radiotherapy treatment rooms are primarily based on photon radiation shielding. Therefore, unaccounted neutron contamination presents a significant challenge for radiation protection. In particular, the dose rates at the maze entrance of the medical linac pose significant radiation protection challenges for workers and the public.

Various studies have been performed to evaluate neutron and gamma capture dose rates at the maze entrance of medical linacs. To determine dose rates at the maze entrance of a medical linac, various empirical formulae have been proposed. However, data on neutron and gamma capture doses for Varian TrueBeam and Elekta Versa HD^TM^ linacs are lacking [14,15]. At the regional level, such studies are important for increasing awareness of radiation protection in radiation oncology centers.

Herein, we evaluated neutron and gamma capture dose equivalent rates for Varian TrueBeam and Elekta Versa HD^TM^ linacs by combining experimental measurements and empirical calculations to evaluate their significance concerning the radiation protection of workers and the public.

## 2. Materials and Methods

### 2.1. Study Design

Measurements and empirical calculations were conducted to evaluate the neutron and gamma capture dose rates at the maze entrance of the Varian TrueBeam and Elekta Versa HDTM linear accelerators. The linacs operate at 15 MV, 10 MV, and 10 MV flattened filter-free (FFF). The experimental measurements were performed at the King Abdallah Oncology Center in Riyadh, Saudi Arabia.

### 2.2. Experimental Measurements

A neutron survey meter (Berthold Technologies, UK) was used to measure the dose rate from neutrons. Gamma dose equivalent rates were measured using Victoreen 450 area survey meter and reported to have an accuracy of within 10% of the reading. Both survey meters were within their calibration validity.

The experimental measurements were performed using Varian TrueBeam and Elekta Versa HD™ linear accelerators. Both medical linacs were previously calibrated to deliver an absorbed dose of 1 cGy/MU X-ray photons using the reference conditions detailed in the International Atomic Energy Agency (IAEA) dosimetry protocol TRS 398 [16].

During measurements, the linac was set to deliver a dose of 600 MU in one minute at the isocenter, corresponding to a total dose of 6 Gy X-ray photons. At each point, three consecutive measurements were made from which an average value was taken. The measured dose from photoneutrons in Sv·Gy^−1^ photons was then converted to a unit of Sv·h^−1^ using the selected linac dose rate.

**Figure 1:**
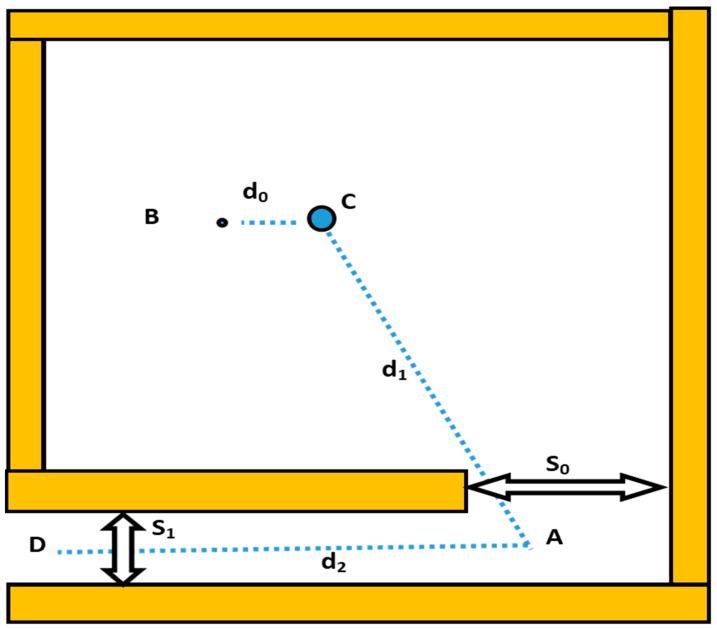
Diagram showing linac vault geometry definitions used for dose calculations. (The point B is at a distance of 1.41 m from the isocenter; d_1_ represents the distance between the isocenter (C) and the just-visible point (A) on the maze centerline; d_2_ is the distance between point A and maze entrance D, where S0 and S1 denote the inner and outer maze entrance cross-sectional areas, respectively).

A patient-equivalent solid water phantom 30 × 30 × 15 cm^3^ was used to simulate conditions similar to patient treatments and provide a similar scatter to that produced in the radiotherapy room. Measurements were made with the gantry pointed vertically upwards, the linac positioned at 180°, after which the collimator closed to provide a minimum field size of 0.5 × 0.5 cm^2^. With the linac operating at its maximum energy, photoneutron and gamma capture dose rates at several points were measured in the treatment rooms using a calibrated neutron detector and a gamma survey meter. All measurements were taken approximately one meter above floor level. Neutron and gamma area meters were placed side-by-side at the point of measurement, following which readings were monitored using a surveillance camera. Figure 1 shows the diagram of a radiotherapy treatment vault with points of dose measurements (A, B, C & D) as the geometric definitions used for dose estimates.

### 2.3. Empirical Methods for Neutron Dose Estimates at the Maze Entrance

#### 2.3.1. Kersey Method

Several methods have been proposed for evaluating neutron dose equivalents at the maze entrance of a medical linear accelerator. The Kersey method was one of the earliest methods to estimate the neutron dose rates at the maze entrance of a medical linear accelerator per unit X-ray absorbed dose at the isocenter ($H_{n,D}$) [17].

(1) $$H_{n,D}=H_{0}\left(\frac{S_{0}}{S_{1}}\right){\left(\frac{d_{0}}{d_{1}}\right)}^{2}10^{-\left(\frac{d_{2}}{5}\right)}$$

where $H_{0}$ is the dose rate measured at point B per Gy of photons at the isocenter. The geometrical parameters *d*_1_*, d*_2_*, S*_0_*,* and *S*_1_ are as defined in Figure 1. This equation is based on the assumption that the maze has a ten-value distance (TVD) of 5 m for the attenuation of neutrons in the maze.

##### Falcão’s Method

Unlike the Kersey method, which assumes a fixed TVD of 5 m (Equation (1)), Falco et al. [18] proposed that TVD values should vary with maze geometry.

(2) $$TVD=1.7+0.55S_{1}$$

$H_{n,D}$ is then estimated using Falcão’s method by replacing TVD with the value in Equation (1).

#### 2.3.2. Modified Kersey Method

Wu and McGinley [19] put forward an equation that considers non-standard surface areas and mazes with exceptional widths or lengths. They proposed a modified Kersey method to empirically calculate neutron dose rates ($H_{n,D}$) at the maze entrance [16].

(3) $$H_{n,D}=2.4\cdot 10^{-15}\varphi_{A}\sqrt{\frac{S_{0}}{S_{1}}}\left(1.64\cdot 10^{-\left(\frac{d_{2}}{1.9}\right)}+10^{-\left(\frac{d_{2}}{TVD}\right)}\right)$$

where $H_{n,D}$ is expressed as Sievert neutrons per Gray photon per m^2^ (Sv·Gy^−1^), and $\varphi_{A}$ represents the neutron fluence at the inner maze entrance per unit absorbed dose of photons (m^−2^·Gy^−1^) at the isocenter, constituting the sum of the direct ($\varphi_{d}$), scattered ($\varphi_{sc}$), and thermal neutron fluence ($\varphi_{th}$) [20].

(4) $$\varphi =\varphi_{d}+\varphi_{sc}+\varphi_{th}$$

or

(5) $$\varphi_{A}=\frac{\beta Q_{n}}{4\pi d^{2}}+\frac{5.4Q_{n}}{2\pi S_{r}}+\frac{1.26Q_{n}}{2\pi S_{r}}$$

where β is a factor that accounts for the transmission through the linac head shielding (1.0, lead and 0. 85 for tungsten), $Q_{n}$ denotes the strength of the neutrons at the isocenter (neutrons per Gy photons), and Sr is the area of the treatment vault (m^2^). In this method, the TVD was determined from the maze geometry as

(6) $$TVD=2.06\times \sqrt{S_{1}}$$

Equations (1)–(6) are valid for determining the neutron dose rates at the maze entrance of a radiotherapy medical linear accelerator operating in the X-ray photon energy range from 6 to 25 MV [6]. Table 1 shows the data and the geometrical information of the two linac vaults used for the dose calculations.

## 3. Results and Discussion

We performed experimental measurements and empirical calculations for the neutron and gamma capture ambient dose equivalent rates at different points of interest in two radiotherapy vaults.

Table 2 illustrates the measurements of the neutron dose rate ($H_{n,D}$) at the isocenter (point C) and points B and A. Shown neutron strength (Q) values are taken from the literature and ranged from 0.02 × 10^12^ for 10 MV to 0.59 × 10^12^ neutrons per Gy photon (n/Gy). At a photon energy of 15 MV, the neutron strength of the Elekta Versa HD is $0.41\times 10^{12}$ n/Gy in comparison to that of the Varian linac’s at $0.59\times 10^{12}$ n/Gy [21,22]. Generally, the linac output is largely affected by the materials in the linac head and the amount of electron energy incident on the target. Furthermore, Varian linacs produce approximately twice as many neutrons as Elekta linacs, which directly affects photoneutron contamination in the linac vaults, since it is attributed to the scatter in the room walls.

In addition to the differences in the accelerator head and collimator materials, differences in the measurement methods may also influence the neutron strength, as can differences in the MC codes and simulated geometries.

### 3.1. Variations of the Neutron and Photon Dose along the Maze

To study the variation in doses towards the maze entrance, we measured the neutron and gamma capture dose equivalent rates at different points along the centerlines of the maze.

**Figure 2:**
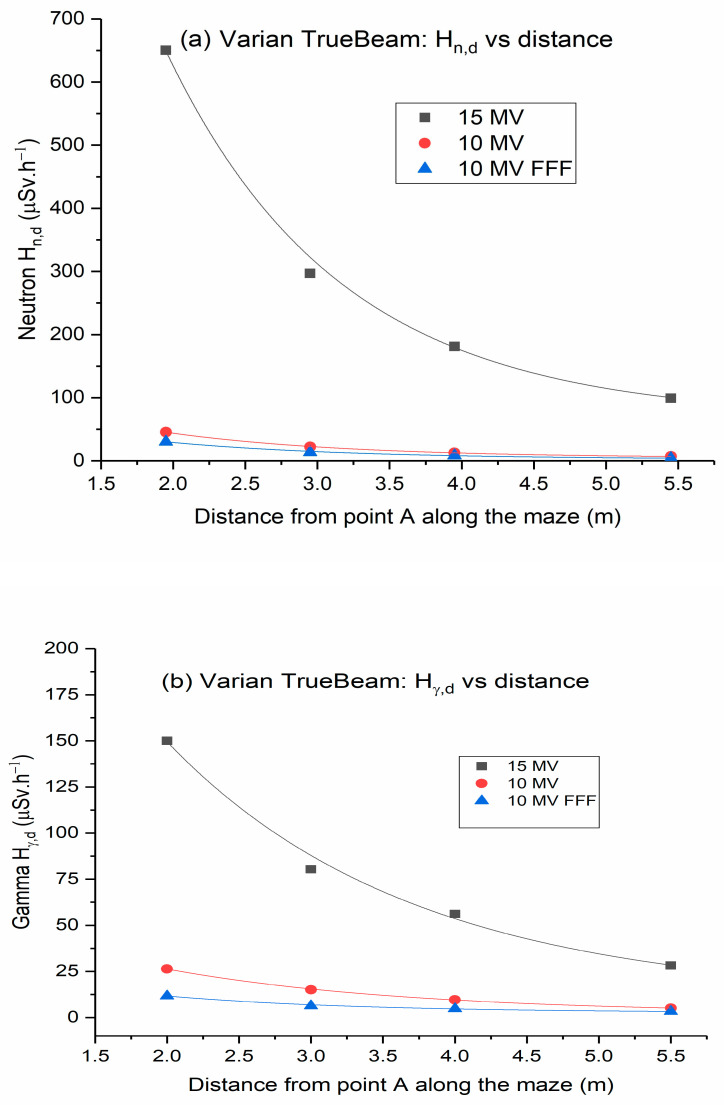
Dose equivalent rates along the maze of Varian TrueBeam linear accelerator: (**a**) neutron dose rates; (**b**) gamma dose rates. (Error bars are not shown because they are too small to be visible in the graph).

**Figure 3:**
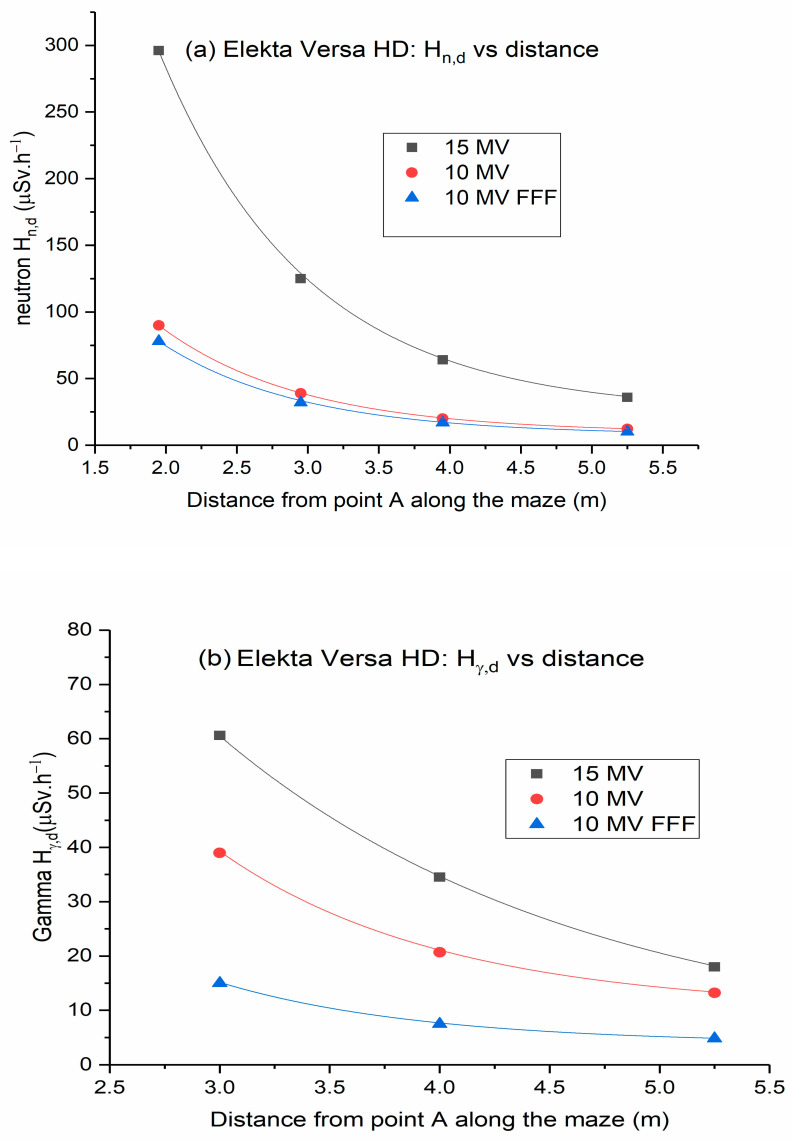
Dose equivalent rates along the maze of Elekta Versa HD linear accelerator: (**a**) neutron dose rates; (**b**) gamma dose rates (Error bars are not shown because they are too small to be visible in the graph).

Figure 2 shows $H_{n,D}$ and $H_{\gamma ,D}$ values plotted against the distance along the maze of a Varian TrueBeam linac. Similarly, Figure 3 illustrates $H_{n,D}$ and $H_{\gamma ,D}$ values plotted against the distance along the maze of Elekta Versa HD linac. As the distance increases toward the maze entrance, the dose of neutrons and gamma decreases exponentially. This could be ascribed to differences in neutron spectra and the nonlinear behavior of the neutron meter near the entrance of the inner maze [19]. Measured gamma doses comprise two components: capture gamma and leakage X-rays. When compared to gamma capture, leakage radiation scattered by walls at points further down the maze was negligible [23,24].

The low-energy scattered X-rays have a short TVD, while the gamma capture component produced through the interactions of the photoneutrons with the linac vault walls tends to have a longer TVD. Consequently, the contribution of the photon dose at the maze entrance comes from gamma capture photons [25,26,27].

### 3.2. H_n_,_D_ and H_γ_,_D_ at the Maze Entrance

Table 3 presents $H_{n,D}$ and $H_{\gamma ,D}$ values at 0.3 m from the maze entrance (µSv·h^−1^) for the three nominal energies. The results are shown for the experimental measurements as well as those calculated using different empirical methods.

Table 3 shows the measured neutron equivalent dose at the maze entrance ranging from 10.2–36.3 µSv·h^−1^ for Elekta Versa HD and 42–99 µSv·h^−1^ for Varian TrueBeam. Our results concur with those reported by Kim et al. [24], who reported $H_{n,D}$ values in the range of 16.4–194 µSv·h^−1^. However, these values are below those presented by McGinley and Miner, 1995, who reported combined neutrons and gamma rays dose-equivalent rates ranging from 2.6 × 10^–7^ to 23.3 × 10^–7^ for different types of maze doors [4]. Expectedly, owing to the increase in photoneutron production in the linac head and the subsequent scattering at the linac vault walls, the total neutron and photon dose rates increased with energy. An important observation from the study of the FFF linac is that it tends to have lower photoneutron contamination owing to filter removal from the linac head [28].

In addition, we determined the radiation at the maze entrance of the two linac vaults using four different common empirical methods reported in the literature (Table 4). While the Kersey method used a conservative approach with a fixed HVD of 5 m in its calculations, as shown in Equation (1), the Falcão and Kersey method used different methods for estimating the TVD. Additionally, using the modified Kersey method shown in Equation (4), doses were estimated using neutron strength (Q), and as we did not calculate Q values in this study, values from the literature were used to calculate $H_{n,D}$ values according to the modified Kersey method. Thus, the results from this method show a wide deviation from those of the other empirical methods used in the present study. Excluding this method, the empirical methods used in this study agree by a factor of 2 or less.

Our results are consistent with those reported by McGinley and Butker in Ref. [23], who evaluated Kersey’s technique for several treatment facilities and several accelerator models reporting a dose ratio of 0.82 to 2.3 between Kersey’s method and the measurements. However, Monte Carlo code simulations can be leveraged as an alternative to the simple empirical methods, which do not consider spectral shape modifications and impact on the neutron energy spectrum weighted dose rate.

### 3.3. Detectors for Secondary Neutron Measurements in Radiotherapy

Neutron detectors are used to measure the absorbed dose or neutron fluence and can be active, thereby providing the dose information in real time or passivity that requires processing to obtain the dose information. Secondary neutrons in radiotherapy are mainly composed of thermal and epithermal neutrons with energies less than 0.5 eV and thermal neutrons with energies between 0.5 eV and 10 keV [2]. For this reason, detector materials are expected to have a high neutron capture cross-section for thermal neutrons. 

Table 4 summarizes common neutron detectors used for secondary neutron measurements in radiotherapy.

This study used a Berthold neutron area survey meter based on a 3He gas proportional counter. Besides this study, various authors have reported the use of neutron survey meters for dose rate measurements in radiotherapy treatment rooms [15,29]. Survey meters based on ^10^BF3 or ^3^He proportional counters are highly stable, have large sensitive areas leading to high signal amplification and tissue equivalence, and exhibit excellent gamma discrimination. However, these instruments suffer from pile-up effects of high photon signal rates, which are bulky and difficult to handle.

Under certain circumstances, passive neutron detectors, such as activation foils and thermoluminescence dosimeters (TLD), may be preferred. A combination of TLD-600/TLD-700 has been used to measure neutron doses in radiotherapy treatment rooms [30,31]. Activation foils have also been used as standalone detectors for neutron fluence measurement [32]. While these are small-sized and have good energy resolution, the induced activity has to be measured in a gamma spectrometer installed away from the measurement location.

Neutron fluence and dose rate measurements have been performed by combining activation foils and TLDs with a Bonner sphere spectrometer (BSS) [33,34]. Other spectrometers, such as a nested neutron spectrometer (NNS), were utilized as well to measure the neutron spectra inside the radiotherapy treatment room [35]. The advantage of neutron spectrometers is that they provide energy information of the neutron spectrum, thus enabling the use of the best fluence-to-dose conversion coefficients for accurate neutron dose estimates. Other common detectors used for secondary neutron measurements in radiotherapy include nuclear tract and bubble detectors.

This study has limitations: we have used a He-3 base Berthold neutron dosimeter known for its pile-up effects at high photon signal rates. As an alternative source was not available to us at the time of measurement, the detector response could not be checked against another neutron source. All these factors may affect the accuracy of our measurements. When feasible, spectroscopic measurements may be more accurate for determining the dose due to photoneutrons.

## 4. Conclusions

Using experimental measurements and empirical calculations, we estimated the neutron and gamma capture dose rates at the maze entrance of two medical linear accelerators. In addition, we measured dose rates at the centerline along the maze towards the maze door to study the impact of maze length on the doses at the maze entrance. The measured total dose equivalent rates were comparable to those reported in the literature. The empirical method for calculating the neutron equivalent dose to the maze entrance agreed within a factor of two from the experimental measurements. The measured neutron and gamma capture doses decreased exponentially towards the maze entrance and were not insignificant for maze lengths less than 6 m. According to the results of this study, the flattening filter-free photon energy tended to have lower photoneutron contamination owing to the removal of filters from the linac head. These findings assume significance to increase awareness of radiation protection in radiotherapy in this region.

## Figures and Tables

**Table 1:** The data and the geometrical information of the two linac vaults.

Geometrical Parameter	Varian TrueBeam	Elekta HD
Energies (MV)	10; 10 FFF; 15	10; 10 FFF; 15
h1 (m)	3.00	3.00
w1 (m)	1.60	1.78
*S*_1_ (m^2^)	4.80	5.34
h0 (m)	3.00	3.00
w0 (m)	1.90	1.90
*S*_0_ (m^2^)	5.70	5.70
*d*_1_ (m)	7.30	7.60
*d*_2_ (m)	5.45	5.25
Room surface area (m^2^)	210.00	210.00

*S*_1_ is the product of the height (h1) and the width (w1) of the outer maze. *S*_0_ is the product of the height (h0) and width (w0) of the inner maze.

**Table 2:** Results of neutron dose equivalent rate measurements at points C, B, and A.

Beam MV	Strength × 10^12^ n/Gy	Delivered X-ray Dose *		Measured H * (10) (µSv/Gy Photons)		
		MU	Gy	C	B	A
Elekta Versa HD						
15	0.42	600	6	48.29	43.03	4.52
10	0.04	600	6	24.96	20.18	1.28
10 FFF	0.02	600	6	20.56	15.40	1.30
Varian TrueBeam						
15	0.59	600	6	72.78	56.72	5.46
10	0.04	600	6	40.95	15.23	0.46
10 FFF	0.03	600	6	31.25	8.12	0.31

* one minute.

**Table 3:** Neutron and gamma dose equivalent at 0.3 m from the entrance of the maze (µSv·h^−1^) per unit absorbed-dose rate (Gy·h^–1^) of X-rays at isocenter (Sv·Gy^–1^).

Beam Energy (MV)	Photon Dose Measurements (µSv·h^−1^)	Neutron Dose Equivalent (µSv·h^−1^)			
		Measurements	Kersey	Modified Kersey	Falcão
Elekta Versa HD					
15	18.00 ± 0.01	36.30 ± 0.00	50.7	158.0	42.0
10	13.20 ± 0.00	12.30 ± 0.00	23.8	15.1	19.7
10 FFF	4.80 ± 0.00	10.20 ± 0.00	16.0	7.5	13.2
Varian TrueBeam					
15	28.20 ± 0.00	99.30 ± 0.03	85.3	211.0	58.2
10	5.10 ± 0.01	69.00 ± 0.05	30.8	13.8	21.0
10 FFF	3.30 ± 0.01	42.00 ± 0.00	14.8	8.6	10.1

**Table 4:** Summary of the common neutron detector used for secondary neutron measurements in radiotherapy.

Detector	Advantages	Disadvantage
3He or BF3 gas-filled proportional counters	3He field detector has a high neutron capture cross-section Large sensitive areas and high amplification Insensitive to gamma	Low thermal neutron cross-section (for BF3-filled detectors) Pile-up effects due to high photon signal rates Bulky and difficult to handle
Solid-state nuclear track detectors	High sensitivity for a wide range of energies Insensitive to gamma Suitable for personnel dose monitoring	Time-consuming track development process and readout procedure Possibility of breakage and scratches
Thermoluminescent dosimeters (TLDS)	TLDs are tissue equivalence and small dimensions Do not need any electronic devices during exposure	Sensitive to high photon flux Need readout system and processing
Bubble detectors	Insensitive to photons, compact, lightweight Have an isotropic response Low-cost and reusable	Limited dynamic range Temperature dependence Energy dependence at high bubble rates
Activation foils	Insensitive to photons Have a small size and low cost Very good spatial resolution	Activity measured in a separate gamma spectrometry No dose information
Bonner sphere systems	Excellent energy range Good neutron sensitivity Good photon discrimination Isotropic angular response	Poor energy resolution Prolonged time required for irradiating the spheres Error-prone spectrum-unfolding process

## Data Availability

Data available on request.

## References

[1] Torre L.A., Siegel R.L., Ward E.M., Jemal A. (2016). Global cancer incidence and mortality rates and trends—An update. Cancer Epidemiol. Biomark. Prev..

[2] Banaee N., Goodarzi K., Nedaie H.A. (2021). Neutron contamination in radiotherapy processes: A review study. J. Radiat. Res..

[3] Barquero R., Mendez R., Vega-Carrillo H.R., Iñiguez M.P., Edwards T.M. (2005). Neutron spectra and dosimetric features around an 18 MV linac accelerator. Health Phys..

[4] Yücel H., Çobanbaş İ., Kolbaşı A., Yüksel A.Ö., Kaya V. (2016). Measurement of photo-neutron dose from an 18-MV medical linac using a foil activation method in view of radiation protection of patients. Nucl. Eng. Technol..

[5] Howell R.M., Ferenci M.S., Hertel N.E., Fullerton G.D., Fox T., Davis L.W. (2005). Measurements of secondary neutron dose from 15 MV and 18 MV IMRT. Radiat. Prot. Dosim..

[6] NCRP (2005). Structural Shielding Design and Evaluation for Megavoltage X- and Gamma-ray Radiotherapy Facilities.

[7] Khan F.M., Gibbons J.P. (2014). Khan’s The Physics of Radiation Therapy.

[8] Mesbahi A., Ghiasi H., Mahdavi S.R. (2010). Photoneutron and capture gamma dose equivalent for different room and maze layouts in radiation therapy. Radiat. Prot. Dosim..

[9] Mobley R.C., Laubenstein R.A. (1950). Photo-neutron thresholds of beryllium and deuterium. Phys. Rev..

[10] Ozerov S., Hagen A.R., Archambault B.C., Sansone A.A., Boyle N.M., Grimes T.F., Rancilio N.J., Plantenga J.M., Taleyarkhan R.P. (2022). Clinical 6 MV X-ray facility photo-neutron/fission interrogations with TMFD sensors. Nucl. Instrum. Methods Phys. Res. Sect. A Accel. Spectrometers Detect. Assoc. Equip..

[11] Attix F.H. (2008). Introduction to Radiological Physics and Radiation Dosimetry.

[12] Pacelli R., Mansi L. (2019). Eric Hall and Amato J. Giaccia: Radiobiology for the Radiologist.

[13] ICRP, International Commission on Radiological Protection (ICRP) (2007). Radiological Protection in Medicine, ICRP Publication 105. Ann. ICRP.

[14] Carinou E., Kamenopoulou V., Stamatelatos I.E. (1999). Evaluation of neutron dose in the maze of medical electron accelerators. Med. Phys..

[15] Wang X., Esquivel C., Nes E., Shi C., Papanikolaou N., Charlton M. (2011). The neutron dose equivalent evaluation and shielding at the maze entrance of a Varian Clinac 23EX treatment room. Med. Phys..

[16] Andreo P., Burns D.T., Hohlfeld K., Huq M.S., Kanai T., Laitano F., Smyth V., Vynckier S. (2004). Absorbed Dose Determination in External Beam Radiotherapy: An International Code of Practice for Dosimetry Based on Standards of Absorbed Dose to Water.

[17] McGinley P.H. (1998). Shielding Techniques for Radiation Oncology Facilities.

[18] Falcao R.C., Facure A., Silva A.X. (2007). Neutron dose calculation at the maze entrance of medical linear accelerator rooms. Radiat. Prot. Dosim..

[19] Wu R.K., McGinley P.H. (2003). Neutron and capture gamma along with the mazes of linear accelerator vaults. J. Appl. Clin. Med. Phys..

[20] IAEA (2006). Radiation Protection in the Design of Radiotherapy Facilities.

[21] Tóth Á.Á., Petrović B., Jovančević N., Krmar M., Rutonjski L., Čudić O. (2017). The evaluation of the neutron dose equivalent in the two-bend maze. Phys. Med..

[22] Maglieri R., Liang L., Evans M., Licea A., Dubeau J., Witharana S., DeBlois F., Seuntjens J., Kildea J. (2014). SU-F-BRE-11: Neutron Measurements Around the Varian TrueBeam Linac. Med. Phy..

[23] McGinley P.H., Butker E.K. (1991). Evaluation of neutron dose equivalent levels at the maze entrance of medical accelerator treatment rooms. Med. Phys..

[24] Kim H.S., Jang K.W., Park Y.H., Kwon J.W., Choi H.S., Lee J.K., Kim J.K. (2009). The new empirical formula for neutron dose level at the maze entrance of 15 MV medical accelerator facilities. Med. Phys..

[25] McGinley P.H., Miner M.S., Mitchum M.L. (1995). A method for calculating the dose due to capture gamma rays in accelerator mazes. Phys. Med. Biol..

[26] McGinley P.H., Dhaba’An A.H., Reft C.S. (2000). Evaluation of the contribution of capture gamma rays, X-ray leakage, and scatter to the photon dose at the maze door for a high energy medical electron accelerator using a Monte Carlo particle transport code. Med. Phys..

[27] Han Z., Chin L.M. (2018). On the tenth value distance of the photon field along the maze of high-energy linear accelerator vaults. J. Appl. Clin. Med. Phys..

[28] Montgomery L., Evans M., Liang L., Maglieri R., Kildea J. (2018). The effect of the flattening filter on photoneutron production at 10 MV in the Varian TrueBeam linear accelerator. Med. Phys..

[29] Rudd P.J., Prior D., Austin-Smith S. (2007). Neutron contamination of 10 MV X-rays: Its relevance to treatment room door and maze design. Br. J. Radiol..

[30] Hsu F.Y., Chang Y.L., Liu M.T., Huang S.S., Yu C.C. (2010). Dose estimation of the neutrons induced by the high energy medical linear accelerator using dual-TLD chips. Radiat. Meas..

[31] Nedaie H.A., Darestani H., Banaee N., Shagholi N., Mohammadi K., Shahvar A., Bayat E. (2014). Neutron dose measurements of Varian and Elekta linacs by TLD600 and TLD700 dosimeters and comparison with MCNP calculations. J. Med. Phys. Assoc. Med. Phys. India.

[32] Followill D.S., Stovall M.S., Kry S.F., Ibbott G.S. (2003). Neutron source strength measurements for Varian, Siemens, Elekta, and General Electric linear accelerators. J. Appl. Clin. Med. Phys..

[33] Esposito A., Bedogni R., Lembo L., Morelli M. (2008). Determination of the neutron spectra around an 18 MV medical LINAC with a passive Bonner sphere spectrometer based on gold foils and TLD pairs. Radiat. Meas..

[34] Bedogni R., Esposito A., Gentile A., Angelone M., Gualdrini G. (2008). Determination and validation of a response matrix for a passive Bonner sphere spectrometer based on gold foils. Radiat. Meas..

[35] Maglieri R., Licea A., Evans M., Seuntjens J., Kildea J. (2015). Measuring neutron spectra in radiotherapy using the nested neutron spectrometer. Med. Phys..

